# Polymorphisms in an intronic region of the myocilin gene associated with primary open-angle glaucoma—a possible role for alternate splicing

**Published:** 2010-12-29

**Authors:** P.J. Eswari Pandaranayaka, N. Prasanthi, N. Kannabiran, K. Rangachari, M. Dhivya, Subbiah R. Krishnadas, P. Sundaresan, S. Krishnaswamy

**Affiliations:** 1 Centre of Excellence in Bioinformatics, School of Biotechnology, Madurai Kamaraj University, Madurai, Tamilnadu, India; 2 Department of Genetics, Aravind Medical Research Foundation, Aravind Eye Hospital, Madurai, Tamilnadu, India; 3 Glaucoma Clinic, Aravind Eye Hospital, Madurai, India

## Abstract

**Purpose:**

To examine the possible role of alternate splicing leading to aggregation of myocilin in primary open-angle glaucoma.

**Methods:**

Several single nucleotide variations found in the myocilin (*MYOC*) genomic region were collected and examined for their possible role in causing splice-site alterations. A model for myocilin built using a knowledge-based consensus method was used to map the altered protein products. A total of 150 open-angle glaucoma patients and 50 normal age-matched control subjects were screened for the predicted polymorphisms, and clustering was performed.

**Results:**

A total of 124 genomic variations were screened, and six polymorphisms that lead to altered protein products were detected as possible candidates for the alternative splicing mechanism. Five of these lay in the intronic regions, and the one that lay in the exon region corresponded to the previously identified polymorphism (Tyr347Tyr) implicated in primary open-angle glaucoma. Experimentally screening the intronic region of the *MYOC* gene showed the presence of the predicted g.14072G>A polymorphism, g.1293C/T heterozygous polymorphism, instead of our predicted g.1293C/- polymorphism. Other than the prediction, two novel SNPs (g.1295G>T and g.1299T>G) and two reported SNPs (g.1284G>T and g.1286G>T) were also identified. Cluster analysis showed the g.14072G>A homozygous condition was more common in this cohort than the heterozygous condition.

**Conclusions:**

We previously proposed that the disruption of dimer or oligomer formation by the C-term region allows greater chances of nucleation for aggregation. Here we suggest that polymorphisms in the myocilin genomic region that cause synonymous codon changes or those that occur in the intron regions can possibly lead to altered myocilin protein products through altered intron–exon splicing.

## Introduction

Glaucoma is a term used to describe a group of disorders that have in common a characteristic degeneration of the optic nerve associated with typical visual field defects and usually elevated intraocular pressure (IOP). If left untreated, the disease progresses to absolute, irreversible blindness. There are different types of glaucoma, depending upon the time of disease manifestation. Juvenile open-angle glaucoma (JOAG) manifests clinically between the ages of 3 and 30 [1,2]. The late onset form of this condition, primary open-angle glaucoma (POAG) usually manifests clinically before the age of 40 and is the most prevalent type [3-5]. The obstruction of the trabecular meshwork—the aqueous humor outflow pathway—is the major cause of the increase in IOP in open-angle glaucoma. It has been reported that mutations in the myocilin (*MYOC*) gene cause POAG [6], which implies a possible role for the product of this gene in the IOP elevation in JOAG. The *MYOC* gene consists of three exons that together encode for 55–57 kDa myocilin protein with 504 amino acids [7,8].

More than a hundred mutations have been associated with POAG and JOAG in studies performed by different groups in various populations (Appendix 1). A plausible model has been built for myocilin protein, to understand the structural basis of the protein and the mutations. To understand the association of these mutations with POAG, the mutations were mapped onto the structural model. The cause of the disease due to these mutants can be either a change in protein conformation resulting from an amino acid change or the production of a shorter peptide due to the generation of a stop codon (stop) such as Arg46Stop, Asp247Stop,  Gln368Stop, Glu483Stop [9-11]. Though synonymous or silent mutations do not cause any amino acid change, such mutations have been reported to be associated with POAG. Although environmentally induced conformational changes remain a distinct possibility, since these mutations cannot be directly correlated with possible protein conformational changes, they become attractive candidates for investigating other possible mechanisms. Single base changes could lead to activation of cryptic splice sites, resulting in aberrant splicing. Such is the case for Hutchinson Gilford progeria syndrome, in which a silent mutation Gly608Gly leads to the activation of a cryptic splice site, resulting in the production of a truncated protein product [12]. The possibility of such an alternative splicing mechanism induced by genomic variations in the human myocilin gene was explored using sequence analysis tools.

## Methods

The model for myocilin was built using a knowledge-based consensus-modeling approach [13,14]. Fold recognition was done using the following Web-based software programs: (1) searches against the protein data bank by position specific iterative BLAST (PSI-BLAST), (2) the Conserved Domain Database, and (3) superfamily (SUPFAM) searches with full-length sequences and overlapping fragments. The SUPFAM search identified a significant match with part of 1h70 (dimethyl arginine dimethyl aminohydrolase, also called pentein) [15]. The 180-to-433 myocilin region was threaded onto A0 through A253 of 1h70 with gaps using the Insight 2000 software. The disulfide bond between Cys245 and Cys437 was identified [16] and found to be feasible in the threading. It was incorporated into the model. For other regions, FASTA searches against the protein data bank were done using overlapping fragments, and significant matches were used as templates for modeling. Myocilin regions 1 to 61, 70 to 174, and 453 to 504 were modeled using 1BOK (445 to 505), 1I84 (S823 to S923), and 1 K8Q (A92 to A816), respectively. The helical segments (34 to 180) were put together, taking into account the secondary structure packing. Consecutive fragments were joined using loop searches with the Insight 2000 software. Splice repair was performed to optimize the peptide geometry using the homology module of the Insight 2000 software. Energy minimization by applying a conjugate gradient algorithm was done using the consistent valence force field until the minimum energy value was obtained. Mutations were mapped onto the model for visualization and interpretation.

To find a possible candidate for alternative splicing, several variations found in the myocilin genomic region were collected and examined for their possible role in causing splice-site alterations. The sequence analysis was done using the GCG software. The genuine splice sites and alternative splice sites from the expressed sequence tag (EST) confirmed splice dataset. The splice datasets were used for the analysis [17]. Multiple alignments of both these sets of splice sites were done using the GCG software. The alignment was done such that the splice-site consensus was retained, that is, with GT in the donor site and AG in the acceptor site. Of the 392 genuine splice sites, 282 (approx 72%) were randomly sampled and used for the multiple sequence alignment. The donor and acceptor sequences were segregated and used separately for creation of multiple alignments. A total of 20 multiple alignments of both donor and acceptor sequences were generated from the sampled set, with each multiple alignment having approximately 15 sequences each. All 209 alternative splice sites were used for the creation of multiple alignments. A total of ten multiple alignments for both the donor and acceptor sequences were created, all of them having approximately 20 sequences each. Thus, a total of 60 multiple alignments were used for profile analysis.

In the original dataset, the splice data were represented with a 70 bp sequence on either side of the splice site. After testing by trial and error, window sizes of 20 bp and 30 bp on either side of the splice site were used for the donor and acceptor sequences, respectively. Using the above-mentioned window sizes, hidden Markov model profiles were created of the 60 multiple alignments and calibrated for use in searching datasets. The single nucleotide polymorphisms (SNPs) in the genomic region of myocilin were collected from the single nucleotide polymorphism database (dbSNP) and from the literature. The base changes corresponding to different SNPs were incorporated into the genomic sequence of myocilin. A dataset of all these sequences was created and used for searching using the GCG software. All the 60 profiles, that is, the 30 donor and 30 acceptor profiles, were used to search the dataset of SNP-incorporated myocilin sequences.

**Figure 1:**
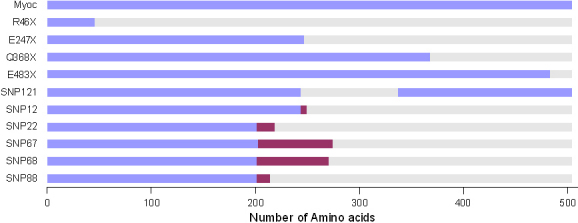
Full length myocilin is  of 504 residues. The stop codon mutations  Arg46Stop, Asp247Stop,  Gln368Stop, Glu483Stop) in mutated myocilin  contains 45 residues,  246 residues, and 367 residues,  482 residues,  respectively and the rest of the region gets deleted.  SNP121 results in shorter protein product containing  410 residues  with amino acids 244 to 337 deleted from full length myocilin protein. SNP12 (rs2032555) consists of 248 residues  with  243 to 248 residues altered from the full length myociln and the rest of the region from amino acid 249 to 504 deleted. SNP22 (rs10690049) has 218 residues with altered amino acids from 201 to 218  and 219 to 504 residues deleted. SNP67 (rs9600235) with 273 residues, 202 to 273 residues altered and 274 to 504 residues deleted. SNP68 (rs11295938) with 270 residues, 201 to 270 residues altered and 271 to 504 residues deleted. SNP88 (rs11366556) with 214 residues, 201 to 214 residues altered and 215 to 504 residues deleted. The maroon colored regions indicate sequence changes introduced because of the possible alternate splicing due to the Single nucleotide polymorphism indicated.

**Figure 2:**
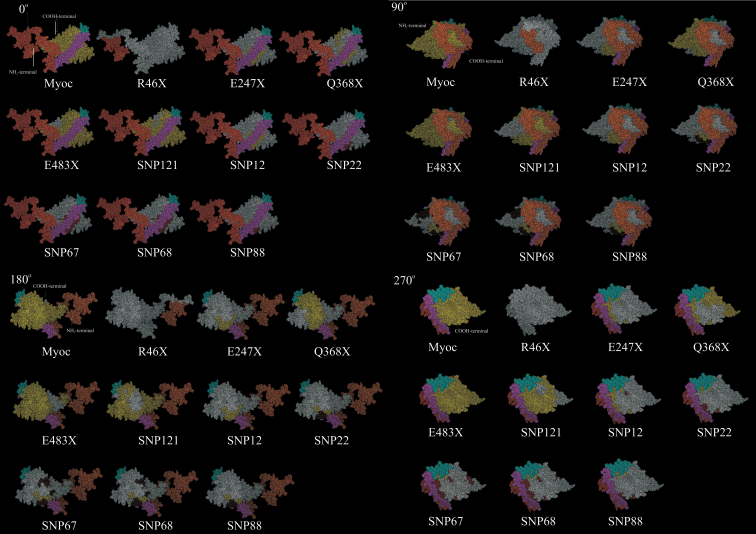
Space-filling model in four orientations for modified myocilin proteins. Full length myocilin, deletions due to presence of stop codon and deletions/modifications due to possible alternative splicing caused by the single nucleotide polymorphism shown in the model, as explained in Figure 1. Different regions in the model are colored as follows; NH_2_-terminal region (Orange), coiled coil region (Pink), hinge region (Cyan), COOH-terminal region (Yellow), regions predicted to be deleted (White) due to stop codon mutation or possible alternative splicing, regions predicted to be modified due to possible alternative splicing.

The alternate splicing of the predicted possible splice sites was theoretically made using GCG by deleting the region that would be deleted if the alternate splicing had occurred. Then the alternate spliced product was translated using the translate command in the GCG. The expected length of the protein product was tabulated (Table 1). Using the same methodology, the product of the deletion mutants due to mutations causing stop codons was obtained. An illustrative bar diagram was made based on the results (Figure 1). These results were also mapped onto the model built to show the possible altered, or truncated, region (Figure 2).

**Table 1:** Possible alternate splice site causing SNP.

**SNP number**	**Genomic location (bp)**	**Location of new site in *MYOC* genome**	**Location of new site in Mutated *MYOC* genome**	**Splice site type**	**Length of altered protein product**	**Known association with disease**
SNP 67 (A>G)	4453 (Intron)	4449	4449	A	215	No
SNP 12 (G>A)	14072 (Intron)	14075	14075	A	258	No
SNP 88 (C/-)	1293 (Intron)	1299	1298	D	214	No
SNP 22 (-/TTTT)	12975 (Intron)	12989	12993	A	225	No
SNP 68 (T/-)	4445 (Intron)	4450	4449	A	275	No
SNP 121 (T>C)	16233 (Exon-3)	16206	16206	A	337	POAG

The genomic location, the location of the splice site in the genome, the location of the splice site in the mutated genome, length of the translated product of the alternate spliced product are shown.

### Case and control study subjects

Unrelated POAG patients of Indian ethnic origin were recruited from the glaucoma services of the Aravind Eye Hospital, Madurai, South India. The study was performed in accordance with its institutional guidelines and the Declaration of Helsinki. Informed consent was obtained from each participant.

Ophthalmic evaluation included best-corrected Snellen visual acuity, measurement of IOP by Goldmann applanation tonometry, anterior chamber angle evaluation by a Goldmann two-mirror gonioscope, and optic disc and retinal nerve fiber examination by a 90-diopter indirect lens. Family history of glaucoma and ocular diseases were also involved in the clinical diagnosis.

Diagnosis of POAG was based on exclusion criteria of congenital glaucoma, angle closure, inflammation, and other secondary causes (trauma, uveitis, or steroid-induced glaucoma). The inclusion criteria were that individuals were diagnosed with POAG, based on the optic disc changes typical of glaucoma, and by matching visual field defects by autoperimetry and open iridiocorneal angles detected by gonioscopy.

Unrelated age-matched control subjects recruited for the study from the general ophthalmology clinic of the Aravind Eye Hospital were diagnosed not to have glaucoma or other major eye diseases. The control population was chosen to match the ethnic and geographic background of the patients with POAG.

### Sample collection and DNA preparation

Our study included 69 JOAG patients, 81 adult onset POAG patients, and 50 normal age-matched control subjects known to be free of glaucoma. The peripheral blood was collected from the patients and control subjects. Genomic DNA was isolated using the salt precipitation method [18]. Briefly the method involves salting out of the cellular proteins by dehydration and precipitation with a saturated sodium chloride solution.

### Polymerase chain reaction

PCR was performed to amplify the specific DNA region corresponding to the predicted alternate splice site causing SNPs of the MYOC gene. Four sets of primers were used, as described in Table 2, using a gradient thermocycler (ASTEC, Fukuoka, Japan). A 20 μl reaction was set up using 50–100 ng of genomic DNA, 1×PCR buffer, 200 µM of dNTPs (Medox Biotech India Pvt. Ltd, Chennai, India), 0.25 picomoles of each primer, and 1 unit of Taq DNA polymerase (Sigma, Saint Louis, MO). The conditions followed were initial denaturation at 94 °C for 3 min, followed by 32 cycles (94 °C for 45 s, 64 °C for 45 s, and 72 °C for 1 min) and final extension at 72 °C for 7 min.

**Table 2:** Primer sequences used to amplify the Specific DNA fragment.

**Primer**	**Primer sequence (5′> 3′)**	**Amplicon size (bp)**
SNP-12-FP	GTCATCCTCAACATAGTCAATCCTTGGGC	440
SNP-12-RP	CAAGTGTGGGTGATAGGATAGAGGGCTTTG	
SNP-22-FP	GCAAAACTGGTCTCAGAAAGGAATCAGACAG	835
SNP-22-RP	GGCTGGTTTGTGAATAGGTGAGTCGTAATTTC	
SNP-67 & 68-FP	GCTTTGGACTGGTCTCCTGTTGAACAGAGCC	798
SNP-67 & 68-RP	GTTTCTCCCCTCACCCTCCCCTCACATCC	
SNP-88-FP	GAGATGGCACCTCTCTGTCAGTTTTCTTAATATG	595
SNP-88-RP	CCTTCGCAACCACAGTATCATTATCTCACCAAG	

### DNA sequencing

PCR Products were extracted from agarose gel and column purified using an EZ-10 spin-column DNA gel extraction kit (Bio Basic Inc., East Markham Ontario, Canada). Bidirectional sequencing was performed using an ABI 3130 Genetic analyzer (Applied Biosystems, Foster City, CA) with dye-termination chemistry.

### Restriction digestion

The PCR amplicon of 440 bp was obtained by using the SNP-12-FP and SNP-12-RP primers digested with Eco72I (Fermentas) to reconfirm the g14072G>A sequence change. Similarly, restriction digestion was performed with HinfI (Fermentas) on the amplified fragment of 798 bp product using using the forward primer SNP-67 & 68-FP and the reverse primer SNP-67 & 68-RP primers to reconfirm the g.4453A>G sequence change.

### Cluster analysis

Cluster analysis was performed using the k-means clustering node of the Clementine software. The MS Excel file with 150 records containing patients’ details such as disease (either POAG or JOAG), age, sex, and g.14072G>A sequence variation was used as input. Using the TYPE node available in the software, the data type of each parameter was defined for further analysis. Then k-means clustering was performed with the five patient detail input categories defined as the optimal “specified number of clusters.” The g.14072G>A polymorphism that we identified in our study was interpreted in these five clusters (Table 3).

**Table 3:** CLUSTER Analysis Data for SNP-12 (g14072G>A).

**Cluster number**	**Number of records**	**Disease**	**Sex**	**SNP-12 (g.14072G>A) GG/GA/AA**
1	46	JOAG	Male	AA (65.22%)
				GA (34.78%)
4	23	JOAG	Female	AA (69.57%)
				GA (30.43)
2	25	POAG	Male	GA (88%)
			Female	GA (12%)
3	41	POAG	Male	AA (100%)
5	15	POAG	Female	AA (100%)

## Results

The NH_2_-terminal region of our predicted myocilin model has less secondary structure content than the other regions in our model. The mid-region forms a set of disjointed helices. This region of disjointed helices can provide flexibility and allow inter-molecular interaction through the coiled coil helical region. The COOH-terminal (181–504) region is quite compact and beta strand rich with the Cys245-Cys433 disulfide bond. Characterization of COOH-terminal olfactomedin-like domain by circular dichroism (CD) has shown that it is predominantly consists of beta-sheet [16]. It contains the olfactomedin-like region (245-504) along with an adjacent 181-244 region. It is identified as the pentein fold of Dimethyl arginine Dimethyl aminohydrolase, which is also reported to form dimers and oligomers [15]. All the mutations are surface exposed. Most of the mutations are surface exposed on the COOH-terminal region [19,20].

Six mutations were detected as possible candidates for having an alternative splicing mechanism (Table 1) when screened for a total of 124 genomic variations. Among the six predicted alternative splice sites causing polymorphisms, one was present in the exon-3 region and the other five were located in the intronic regions. As a result of the theoretical alternate splicing, the protein product obtained after translation had altered regions and deleted regions due to the alternate splicing. The protein products were shorter than full-length myocilin, which consists of 504 amino acids (aa). The stop codon mutations (R46X, E247X, Q368, and E483) seen in the mutated myocilin sequence contained 45 aa, 246 aa, 367 aa, and 482 aa, respectively, and the rest of the region was deleted. The details are shown as a bar diagram in Figure 1. A view of the region being deleted or altered in the model is given in Figure 2.

### Screening for the predicted intronic SNPs

**Figure 3:**
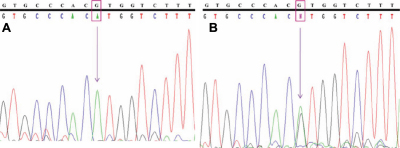
Chromatogram of *MYOC* showing predicted splice site variation (**A**) homozygous g.14072G>A in patient-1 and (**B**) heterozygous g.14072G>A in patient-2. Top line: wild type sequence. Bottom line: observed sequence. The arrow indicates the position of sequence variation. Box represents the variation.

**Figure 4:**
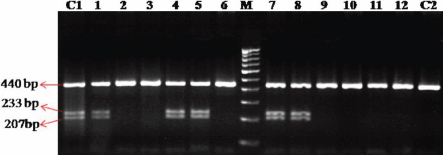
Eco72I restriction digestion to reconfirm the predicted g.14072G>A polymorphism. 1,4,5,7,8: POAG samples showing three distinct bands of 440bp, 233bp and 207 bp (reconfirms the heterozygous g.14072G>A polymorphism. 2,3,6,9,10,11,12: POAG samples showing a distinct band 440 bp (reconfirms the homozygous g.14072G>A polymorphism). C1: Control sample showing heterozygous g.14072 G>A polymorphism. C2: Control sample showing homozygous g.14072 G>A polymorphism. M: 100 bp DNA ladder. Arrow indicates the product size.

In our study, open-angle glaucoma patients had been screened for the presence of such predicted intronic SNPs. We found one of the predicted g.14072G>A (IVS2+35G>A) polymorphisms (rs2032555) in all the 150 patient samples and also in the 50 control subjects with different frequencies of homozygous (AA) and heterozygous (GA) states (Table 4). The homozygous and heterozygous conditions of the g.14072G>A polymorphisms of a patient sample are showed in the Figure 3 chromatogram. This sequence variation was reconfirmed by restriction digestion analysis with Eco72I. The sequence change lacked the Eco72I site and showed a distinct band of 440 bp (mutant allele) with a homozygous condition, whereas in the heterozygous condition, it showed three distinct bands of 440 bp, 233 bp, and 207 bp after digestion (Figure 4). The wild type should show two distinct bands of 233 bp and 207 bp, which were not found in this study.

**Table 4:** Genotype frequency for the predicted *MYOC* polymorphisms.

			Genotype frequency (%)*	
**Location**	**Polymorphism**	**Genotype**	POAG n=150 (%)*	Control n=50 (%)*
Intron-2	SNP-12 (g.14072G>A)	GG	0 (0)	0 (0)
		GA	47 (31.3)	18 (36)
		AA	103 (68.7)	32 (64)
Intron-1	SNP-22 (g.12975TTTT/-)	TTTT/-	0 (0)	0 (0)
Intron-1	SNP-67 (g.4453A>G)	AA	150 (100)	50 (100)
		AG	0 (0)	0 (0)
		GG	0 (0)	0 (0)
Intron-1	SNP-68 (g.4445T/-)	T/-	0 (0)	0 (0)
Intron-1	SNP-88 (g.1293C/-)	CC	145 (96.6)†	50 (100)
		C/-	0 (0)	0(0)

The asterisk represents the genotype frequency in brackets. The dagger indicates that out of 150 POAG, the rest 5 samples showed the g.1293 C>T sequence variation (data was included in Table 5).

**Figure 5:**
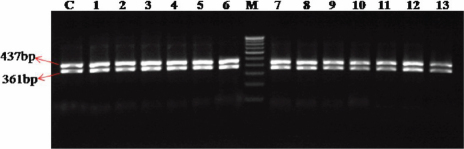
HinfI restriction digestion to screen the g.4453A>G polymorphism. 1 to 13 : Patient samples showing two distinct bands of 437 bp, 361 bp (absence of predicted g.4453A>G polymorphism). C: Control sample. M: 100bp DNA ladder. Arrow indicates the product size.

The predicted g.4453A>G polymorphism (rs9660235) was not identified in our study subjects (patient and control subjects). This was reconfirmed by restriction digestion of specified PCR fragments with HinfI. The wild type showed two distinct bands of 437 bp and 361 bp due to the presence of the HinfI site. The g.4453A>G sequence change should show a 798 bp product in the homozygous condition, and the heterozygous condition of this should show three distinct bands of 798 bp, 437 bp, and 361 bp, which were not identified in the patient nor in the control subjects. Both patient samples and control samples showed two bands of 437 bp and 361 bp product (Figure 5), which confirms the absence of g.4453A>G polymorphism (Table 4).

Among the five predicted intronic polymorphisms, the g.1293C/- (rs11366556), g.4445T/- (rs11295938), and g.12975TTTT/- (rs10690049) were deletion polymorphisms which were not identified in the present study population.

### Other polymorphisms identified

**Figure 6:**
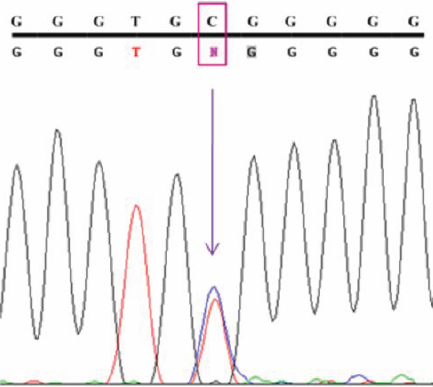
DNA sequence from Intron-1 of *MYOC* showing the g.1293C>T polymorphism. Top line: wild type sequence. Bottom line: observed sequence. The arrow indicates the position of sequence variation. Box represents the variation.

We identified five polymorphisms other than the six predicted polymorphisms (Table 5). Instead of our predicted g.1293C/- polymorphism, we identified a g.1293C/T heterozygous polymorphism in five of the patient samples (Figure 6). This variation was not shown in any of the control samples.

**Table 5:** Genotype frequency for the identified *MYOC* polymorphisms (other than predicted polymorphism).

			Genotype frequency (%)*	
**Location**	**Polymorphism**	**Genotype**	POAG n=150 (%)*	Control n=50 (%)*
Intron-1	g.1284G>T	GG	6 (4)	0 (0)
		GT	0 (0)	6 (12)
		TT	144 (96)	44 (88)
Intron-1	g.1286G>T	GG	6 (4)	0 (0)
		GT	0 (0)	6 (12)
		TT	144 (96)	44 (88)
Intron-1	g.1293C>T	CC	145 (96.6)	50 (100)
		CT	5 (3.3)	0 (0)
		TT	0 (0)	0 (0)
Intron-1	g.1295G>T	GG	141 (94)	47 (94)
		GT	9 (6)	3 (6)
		TT	0 (0)	0 (0)
Intron-1	g.1299T>G	TT	6 (4)	0 (0)
		TG	0 (0)	6 (12)
		GG	144 (96)	44 (88)

The asterisk represents the genotype frequency in brackets.

The g.1284G>T (rs2901559), g.1286G>T (rs2901558), g.1295G>T, and g.1299T>G sequence variations were also identified with different frequencies in the POAG samples and control samples (Table 5).

The screening results showed that, in JOAG patients, the g.14072G>A homozygous polymorphism was seen at a frequency of 65.22% in males and in females at 69.57%; whereas the g.14072G>A heterozygous condition was seen at 34.78% in males, and a genotypic frequency of 30.43% was seen in females. POAG patients had a g.14072G>A homozygous condition at a 65.07% frequency in males and an 83.33% frequency in females. The g.14072 heterozygous state was seen in 34.92% of the males and in 16.66% of the females.

### Cluster analysis for SNP-12 (g.14072G>A)

With the analysis of SNP12, clusters 1 and 4 showed frequencies of g.14072G>A polymorphisms with homozygous and heterozygous states in male and female JOAG samples. Cluster 2 showed the frequency of g.14072G>A polymorphisms with heterozygous states in male and female POAG samples. Cluster 3 and cluster 5 showed the frequency of g.14072G>A. polymorphisms with homozygous conditions in male and female POAG samples (Table 3).

## Discussion

Among the six predicted mutations, one mutation has previously been reported to be associated with POAG—the Tyr347Tyr mutation (Table 1). The Tyr347Tyr silent mutation results in a single base change T>C in exon 3 of myocilin. In a study done on POAG patients from five different populations [9], this mutation showed a high frequency of occurrence, that is, 4.6%, which was considerably higher than most of the other detected mutations used in that study. Thus, this mutation becomes a likely candidate for experimental validation to confirm the role of alternate splicing in POAG. The remaining five mutations are found in the intronic region of myocilin and have not been associated so far in any case of open-angle glaucoma (Table 1).

After incorporation of the six identified splice sites into the myocilin genomic sequence, as expected, the predicted protein products were truncated for all these mutations (Figure 1). Such products may result in altered myocilin protein in which the COOH-terminal oligomer formation would be disrupted resulting in protein aggregates. This helps to explain the characteristic increase in IOP during open-angle glaucoma due to accumulation of protein aggregates in the trabecular meshwork of the eye. The view that disease-causing mutations in myocilin could be a gain of function [21] also supports the implication that altered protein products lead to POAG.

The location of these mutations in the model, the truncation of the protein due to mutations causing stop codons, and the altered protein products due to possible alternative splicing (Figure 2) suggest that a plausible mode of action could be by disruption of dimer or oligomer formation by the COOH-terminal region or conformational changes of the NH_2_-terminal. Hinge regions induced by the molecular environment in the normal protein could also favor aggregation. This would also explain why only 2%–4% of POAG are associated with mutations [19,20]. The Tyr347Try polymorphism implicated in POAG cannot be understood in terms of conformational changes, as the amino acid is not modified. However, the implication from this study that the SNP leading to the Tyr347Tyr polymorphism could lead to alternate splicing resulting in an altered protein product is a possible hypothesis that can be experimentally verified. Identification of such possible polymorphisms that can cause splice-site variations, especially in the intron regions, has not been screened so far. This analysis suggests that it will be necessary to look for the occurrence of such SNPs (especially in intron regions) that are likely to create putative alternate splice sites in open-angle glaucoma patients and also to experimentally validate the presence of alternate spliced products.

This study looked for occurrences of the predicted SNPs; g.14072G>A sequence variation was observed in both homozygous and heterozygous states. This polymorphism was reported as a common polymorphism with the frequency of 47.83% in Iranian JOAG patients with both homozygous and heterozygous conditions [22]. This SNP was also reported as the most common polymorphism, with the frequency of 31.3% (p =0.77) in a Chinese cohort [23]. Our study shows that the g.14072G>A polymorphism is most frequently harbored in the Indian population.

The g.4453A>G polymorphism was observed in 8 unrelated adult African Americans (4 male and 4 female) enrolled in Houston, TX (Trace data was generated by Whitehead Institute for Biomedical Research through a grant from NIH. Whitehead Institute/MIT Center for Genome Research, Sanger Institute, UK and NHGRI, NIH). The 8 samples were derived from the Baylor Polymorphism Resource which includes >500 ethnically diverse samples used as controls (unpublished data). As there are no other reports or frequency data available, this SNP is not known to be present in any other population. The g.4453A>G polymorphism was not identified in the present study population also.

The g.1293C/- deletion polymorphism was another prediction of this study. Though it was observed in eight people of African-American descent from the Baylor Polymorphism Resource, this SNP was not identified in our study subjects.

One thymidine base-pair deletion in the g.4445T/- polymorphism and four thymidine (TTTT) base-pair deletions in the g.12975TTTT/- polymorphism should have been identified according to the prediction. In direct sequencing we could not identify these two variations in our study subjects.

Other than the six predicted polymorphisms, in the present study we identified five other polymorphisms (Table 5). Among them, one was identified as a g.1293C/T heterozygous polymorphism, which was located in our predicted g.1293C/- polymorphic site. This g.1293C/T heterozygous polymorphism was observed in five of the patient samples (Figure 6). Out of five samples, four harbored the predicted g.16233T>C (c.1041T>C) polymorphism in exon-3, which results in the Tyr347Tyr silent mutation associated with POAG. The remaining sample was from a 30-year-old JOAG patient carrying a homozygous g.14072G>A polymorphism. Since the g.1293C/T polymorphism has not been reported elsewhere, we considered it a novel polymorphism. Myocilin accounted for about 2%–4% of the gene mutations in POAG patients [20]. The g.1293C/T heterozygous polymorphism, observed at a 3.3% frequency, was not identified in any of the control samples that could be associated with the disease. Further studies with a large number of sample sizes from various ethnic groups and functional studies are necessary to identify the disease association with this polymorphism.

**Figure 7:**
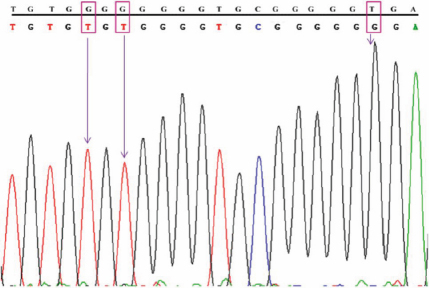
Chromatogram representing the g.1284G>T, g.1286G>T, and g.1299T>G polymorphisms from Intron-1 of *MYOC*. Top line: wild type sequence. Bottom line: observed sequence. The arrow indicates the position of sequence variation. Box represents the variation.

**Figure 8:**
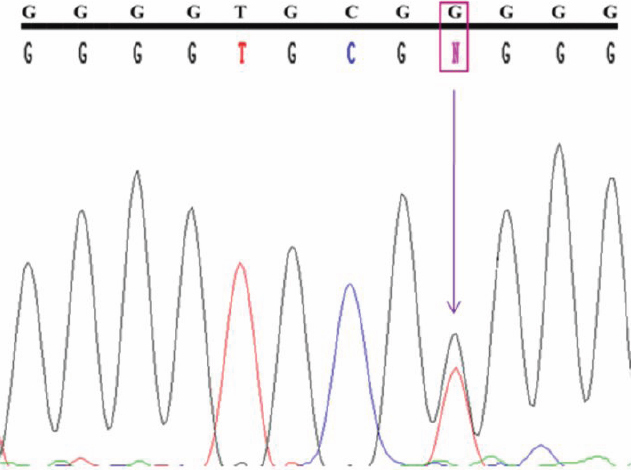
DNA sequence from Intron-1 of *MYOC* showing the g.1295G>T homozygous polymorphism. Top line: wild type sequence. Bottom line: observed sequence. The arrow indicates the position of sequence variation. Box represents the variation.

Among the other four sequence variations, the homozygous g1284G>T, g1286G>T, and g1299T>G polymorphisms were commonly present in the study subjects (Figure 7). The heterozygous g1295G>T sequence change (Figure 8) was observed with less frequency in POAG samples and the control subjects (Table 4). To our knowledge, the g1295G>T and g1299T>G variations were novel polymorphisms that were seen in both POAG samples and in the control subjects.

The cluster analysis results showed that the g14072G>A homozygous polymorphism were more common than the g14072G>A heterozygous polymorphism in both JOAG and POAG patients (Table 3). In an Iranian JOAG population, the g14072G>A homozygous polymorphism was also reported at a slightly higher frequency than the g14072G>A heterozygous polymorphism [22]. However, this condition may vary from one ethnic group to the other. Further studies have to be done with a larger number of samples to determine which age groups are more prone to having the g14072G>A polymorphism.

The presence of truncated or altered mRNA products also needs to be experimentally verified. The computational and experimental analysis in this study has provided a new line of experiments for research in POAG.

## Appendix 1. Mutations associated with open angle glaucoma.

To access the data, click or select the words “Appendix 1.” This will initiate the download of a Microsoft Word document (.doc) that contains the file.
